# Multicomponent exercises to prevent and reduce back pain in elderly care nurses: a randomized controlled trial

**DOI:** 10.1186/s13102-022-00508-z

**Published:** 2022-06-21

**Authors:** Ann-Kathrin Otto, Bettina Wollesen

**Affiliations:** 1 Department of Human Movement Science, University of Hamburg, Turmweg 2, 20148 Hamburg, Germany; 2 Department of Biopsychology and Neuroergonomics, Technical University of Berlin, Fasanenstraße 1, 10623 Berlin, Germany

**Keywords:** Elderly care nurses, Multicomponent intervention, Low back pain, Prevention

## Abstract

**Background:**

Sports science is making an important contribution to health services research and supports the development of tailored interventions, e.g., in nursing settings. Working in elderly care is associated with a high prevalence of low back pain (LBP). Due to the diverse requirements and high strains, multicomponent programs are essential to address all relevant factors. This randomized controlled trial investigated the effects of a tailored ten-week ergonomics and twelve-week strength training on lifting behavior, strength endurance, LBP, functional impairment and adherence.

**Methods:**

n = 42 nurses were randomly assigned to the intervention (IG; n = 20) or control group (CG; n = 22). They were eligible for participation if they were active in residential care and if they provided written informed consent. Other employees were excluded. The data were collected at baseline, at ten weeks (after ergonomics training), at 22 weeks (after strength training), and at 34 weeks (follow-up). The analysis combined physical tests with questionnaires (Progressive Isoinertial Lifting Evaluation, PILE-Test; Biering-Sørensen-Test; Visual Analog Scale Pain, VAS; Oswestry Disability Index, ODI; self-developed questionnaire for adherence). Group differences were analyzed by Chi^2^-Tests, ANOVA, and Linear Mixed Models.

**Results:**

The IG showed an improved lifting performance (PILE-Test; 95% CI 1.378–7.810, *p* = .006) and a reduced LBP compared to the CG (VAS; 95% CI − 1.987 to 0.034, *p* = .043) after ergonomics training (PILE-Test, F_(1,34)_ = 21.070, *p* < .001; VAS, F_(1,34)_ = 5.021, *p* = .032). The results showed no differences concerning the Biering-Sørensen-Test and the ODI. Positive adherence rates were observed.

**Conclusions:**

This approach and the positive results are essential to derive specific recommendations for effective prevention. The study results can be completed in future research with additional strategies to reduce nurses’ burden further.

***Trial registration*:**

The trial was registered at DRKS.de (DRKS00015249, registration date: 05/09/2018).

## Introduction

Increasingly, sports science is making an essential contribution to health services research, addressing occupational health management and promotion in various settings, such as nursing. An exercise science approach that complements public health research supports the development of tailored interventions and thus increases the likelihood of positive effects due to targeted physical adoptions [1, 2].

Health promotion is of particular relevance in the care of the elderly due to the increasing number of multimorbid people in need of care, resulting in a high physical and psychological strain which is associated with a high prevalence of LBP.

The high occurrence, incidence, and recurrence of LBP are caused by multiple factors. Therefore, ergonomic, social, biological, psychological and environmental factors such as e.g., lack of social prestige, work shift organization, the number of staff members on duty, psychological stress, and age play a crucial role [1–3]. However, physical workload, awkward work posture, frequent resident handling activities such as manual lifting, and low physical capacity of the nurses have been identified as the most affecting factors for LBP [4–6]. The demanding physical requirements in accumulated nursing work shifts resulted in changes in physical function with a decline of explosive and maximal strength [7, 8]. Consequently, muscular fatigue could lead to work-related musculoskeletal disorders, suggesting that there is a need for strengthening training [8]. Furthermore, the already existing shortage of skilled workers further aggravates the situation, putting more workloads on the existing staff [9]. Therefore, the relevance of workplace health promotion programs for this target group is of utmost interest to secure the work capacity in this field.

Although the number of health promotion programs in the nursing sector is increasing [10–13], the evidence of successful interventions affecting LBP in elderly care is weak and heterogeneous [12, 14–17].

For example, multicomponent interventions investigated the effectiveness of participatory ergonomics, physical training, and cognitive-behavioral training [14–16] and found improved lifting performance [16] but failed to show effects on perceived muscle strength [14], LBP [14, 16] or working posture [16]. While Rasmussen and colleagues [15] reduced LBP in nursing aides, there is no evidence for any intervention affecting LBP in elderly care.

Reasons for the weak evidence might be setting-specific factors like organizational barriers, time pressure, shift work, and staff shortages, resulting in low attendance and adherence [18, 19]. A key factor for the successful implementation of interventions is proven to be the involvement of employees in the planning and implementation [20]. Thus, the differentiated documentation of work-related risks, wishes, needs, and barriers is relevant to ensure the initialization of sustainable and long-term behavioral modifications and maintain these employees’ motivation [20, 21]. Due to the expected work of nurses, accompanied with burdens, multicomponent interventions with ergonomics and strength training, especially, are required to consider all relevant factors. Based on these previous study results, the BASE concept, a multimodal approach for health promotion developed in Germany (BASE: B ‘Bedarfsbestimmung’ (requirements), A ‘Arbeitsplatzorganisation’ (organization of work), S ‘Schulung des arbeitsbelastungsverträglichen Alltagshandelns’ (coaching preventive behavior at work) and E ‘Eigenverantwortung und Selbstwirksamkeit’ (self-responsibility and self-efficacy)) might be efficient to reduce LBP in the nursing setting [20]. BASE, including ergonomic training, was successful, for example, in a logistics department, reducing LBP and decreasing dysfunctional lifting behavior. Furthermore, the concept indicates an increased motivation for further strength training [20].

Therefore, this randomized controlled trial aimed to investigate whether an intervention program combining ergonomics training with strength and resistance training tailored to the target group improves lifting behavior, strength endurance, LBP, and functional impairment caused by back pain.

The main research question was

- Which effects of the Multicomponent intervention (combination of ergonomics and strength training) related to LBP, functional impairment, lifting behavior, and strength endurance of the lumbar extensors of elderly care nurses can be demonstrated?

We hypothesized significant improvements in lifting behavior, strength endurance and reductions in LBP, and functional impairment in elderly care nurses for the combination of the different parts of the program. We suppose that the combination of both training contents is more beneficial than only preventive ergonomics training to gain positive results.

Moreover, we evaluated the adherence to the intervention program to control if the intervention is feasible to motivate the participation of the elderly care nurses.

## Methods

The CONSORT statement (updated guidelines for reporting parallel group randomized trials) [22] was used as a guideline to report this randomized controlled trial.

### Study design

This crossover single blind randomized controlled trial (RCT) was conducted in two nursing homes (Germany, September 2018 -September 2019). The study is part of the project ‘PROCARE-Prevention and occupational health in long-term care’ [23].

The investigation was approved by the local ethics committee (University of Hamburg, AZ:2018_168) and is registered at DRKS.de (DRKS00015249, registration date: 05/09/2018). Participation in this study was voluntary. The study followed the principles of the Declaration of Helsinki.

### Participants and recruitment

A sample size calculation (G*Power; Version 3.1.9.2, Heinrich Heine University of Duesseldorf) with an estimated effect size of f = 0.25, α = 0.5, and 1 − β = 0.95 for repeated-measures design revealed a number of N = 36 participants. Furthermore, we estimated a dropout rate of 30% and therefore included a total number of N= 45 participants.

All nurses (N = 212) of two nursing home facilities were asked to participate in comprehensive information events by the study director and through flyers distributed by the respective manager in the team meeting. Interested participants signed up for a list with scheduled assessment sessions.

A total of n = 68 nurses and nurse aides agreed to participate. Nurses were included if they were active in residential care and provided written informed consent. Other employees like e.g., home management, and psycho-social carers were excluded. We applied no other inclusion or exclusion criteria.

### Randomization and assignment to the intervention

**Fig. 1:**
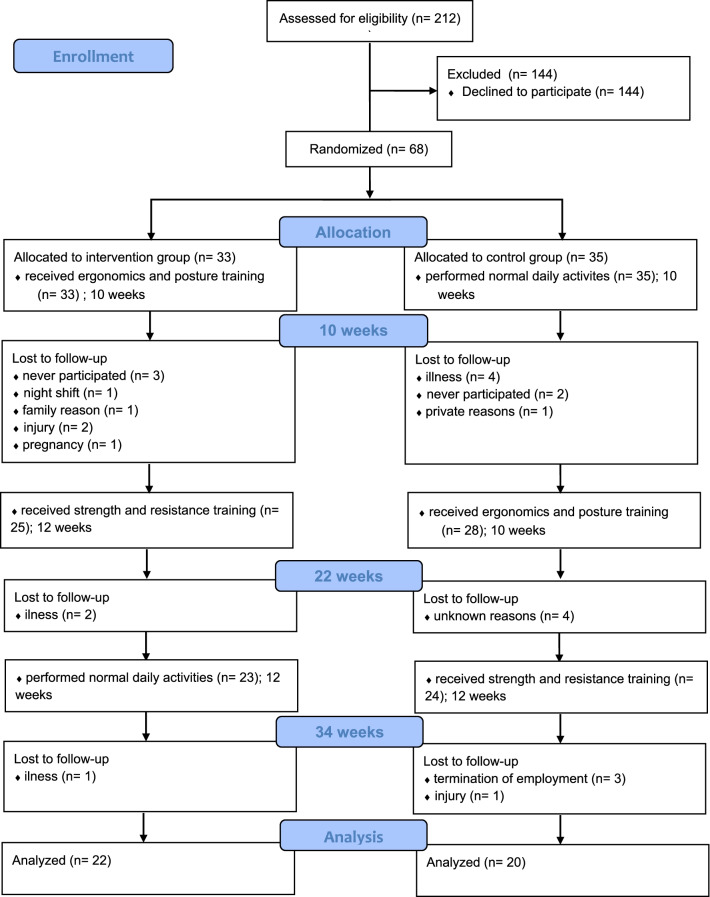
Flow diagram

The random allocation sequence to the IG or the wait-list CG was done manually by lot to avoid selection bias. The random assignment was conducted after the baseline assessment by the study director, who was not involved in intervention procedures. The data were assessed at baseline (pre-test), at ten weeks (post-test 1; IG: after ergonomics training, CG: after normal daily activities), at 22 weeks (post-test 2; IG: after strength training, CG: after ergonomics training), and 34 weeks after starting the program (follow-up; IG: after normal daily activities, CG: after strength training) (Fig. 1). Data collection was done by blinded assessors in a strictly pseudonymized form to guarantee a blinded analysis (e.g., NN08ER30). Following the data collection, participants were informed regarding the group allocation and the study flow. The IG and CG performed the same Multicomponent intervention, starting at different times. The groups were described to the participants as the immediate starting group (means the IG) and the delayed starting group (means the CG). As the study was conducted as a cross-over design, allocation concealment was not necessary.

Nurses were excluded from analysis when the participation rate in the intervention program was below 70%. Of the 68 participants, there were 26 dropouts, which corresponds to a dropout rate of 38%. In the IG, eleven nurses dropped out due to illness (n= 3), injury (n= 2), pregnancy (n= 1), night shifts (n= 1), or family reasons (n= 1). Three individuals never participated in the intervention. Of the 15 dropout individuals in the CG, four were due to illness, another participant due to injury, one due to personal reasons, three due to the termination of the employment relationship, and two never participated in the intervention. For the last four, the reasons were unknown. Overall, 24% of the participants in IG and 37% in CG dropped out during the intervention period.

In total, the analysis included 42 nurses with a mean age of 42.5 (SD ±10.5, 92.9% female). Subject characteristics of the participants and dropouts are presented in Table 1. The participants were analyzed in their original assigned groups. There were no significant differences in baseline characteristics between the IG and the CG. Furthermore, participants lost to follow-up were not significantly different from the considered.

**Table 1:** Baseline characteristics of participants and dropouts

	Intervention (n = 22)	Control (n = 20)	Stat. analysis			Dropout (n = 26)	Stat. analysis		
			F(1,40)	p	ɳ2p		F(1,66)	p	ɳ2p
Age, Mean (SD) (years)	41.1 (10.5)	44.0 (10.7)	.820	.371	.020	44.7 (11.8)	1.156	.286	.017
Female, n (%)	22 (100)	17 (85.0)	–	–	–	23 (88.5)	–	–	–
Height, Mean (SD) (cm)	165.8 (7.7)	165.9 (7.5)	.001	. .980	.000	165.0 (7.5)	.175	.677	.003
Weight, Mean (SD) (kg)	73.2 (12.8)	81.4 (18.6)	2.813	.101	.066	73.6 (21.0)	.552	.460	.008
VAS (score)	1.2 (1.6)	2.1 (2.2)	2.195	.146	.052	2.2 (3.1)	.654	.422	.010
ODI (%)	7.8 (6.2)	12.8 (9.9)	3.623	.064	.083	13.6 (13.5)	1.962	.166	.029

*VAS* Visual Analog Scale, *ODI* Oswestry Disability Index

### Outcome measurements

The Progressive Isoinertial Lifting (PILE-Test), the endurance of the trunk extensor muscles (Biering-Sorensen-Test), and the LBP (VAS) were assessed as primary outcomes.

#### PILE-Test (Progressive Isoinertial Lifting Evaluation)

The PILE-Test recorded the nurses' lifting performance, including psychophysical fatigue of the trunk and extremities. The nurses repeatedly lifted a weight in a box from the floor at the level of their trochanter major and placed it on a nursing bed. The weight was increased by 2.5 kg after an interval of four lifting attempts in 20 s, starting with a total weight of 4 kg for women and 6 kg for men. The test stopped when the nurse exceeded the 20-second interval, decided to quit due to muscular fatigue or pain, reached 85% of the maximal heart rate (220 - age), or when the maximum weight that can safely be lifted has been reached (50% of body weight). Following the suggestion by Wollesen and colleagues [24], additional criteria were added for recording dysfunctional posture: Thoracic spine hyperkyphosis or lumbar spine kyphosis and activation of additional musculature. The weight that the participants could lift in the given time without a termination criterion was recorded. The relative test-retest reliability was high (ICC= 0.91) [25].

#### Biering-Sørensen-Test

The Biering-Sørensen-Test measured the strength endurance of the lumbar extensors. The nurses lay prone on a padded massage table. The body’s trunk extended off the edge of the table at the anterior superior iliac spine level. The buttocks and ankle joints were fixed to the table with straps. At the start of the test, the subject raised his upper body to a horizontal position, crossing their arms in front of their chest, holding their head in a neutral position, and looking down to the floor. Then, the participants were introduced to hold the position until the maximum duration time of 240 s ends or if they leave the test situation due to fatigue. Another termination criterion was the deviation of the horizontal line by more than 5%, measured with a stadiometer [26]. The time that the participants remained in the position without occurring a termination criterion was recorded. The reliability shows ICC values of 0.77–0.83 [27].

#### Visual analog scale (VAS)

The Visual Analog Scale measured the subjective perception of LBP intensity. The scale is a horizontal line anchored by smileys at each end. The smileys ranged from no pain (0) to very severe pain (10). The participants marked the point that they feel represents their perception of their current state. The test-retest reliability is ICC= 0.95 [28].

The Disability Index due to LBP (Oswestry Disability Index) and adherence were assessed as secondary outcomes.

#### Oswestry disability index

The Oswestry Disability Index measured functional impairment caused by back pain during nine different daily activities: personal care, lifting, walking, sitting, standing, sleeping, sex life, social life, and traveling. The scale ranged from 0 to 5, with higher values representing more significant disability. The functional impairment caused by back pain is then calculated using the score obtained divided by the maximum possible score x 100. The minimum value is 0% (no disability) and the maximum value is 100% (bedridden). The correlation coefficient for test-retest reliability is ICC= 0.96 [29].

#### Adherence

The adherence was measured by a list where the attendance of the participants and reasons for dropout were documented and by a self-developed questionnaire [20]. The participants rated twelve questions, for example: 'I liked the exercises', 'I was able to execute the exercises, or 'The exercises were related to my everyday work'. The response options were presented on a 3-point scale, ranging from 1 (Yes, I agree) to 3 (No, I disagree). The responses were presented as frequencies of agreement.

Additionally, demographic characteristics were collected, such as gender, age, body height, and body weight.

### Intervention

The intervention program consisted of standardized ergonomics training and standardized strength training [20, 30]. The program followed the validated BASE concept and workplace observations in each facility. The intervention started with observing daily nursing routines in each facility to identify specific primary ergonomic conditions (e.g., existing lifting aids). Work-related tasks were observed to determine areas where nurses have to assume awkward postures or are exposed to particularly high physical workload (e.g., during residents positioning). In addition, nurses' health risks, wishes, needs, and barriers were surveyed before as part of the PROCARE project and considered for planning and implementing them in training.

The ergonomics training took place over a period of ten weeks (once a week for 20–30 min) with six to eight participants per group. In order to deal with physical stress at the workplace and compensate for it, the training included learning different techniques and compensatory exercises (Table 2). Each unit prioritized different work-related tasks and topics (e.g., work organization, working on care bed, transfer situations). The units included the results of the workplace observation (e.g., handling of existing lifting aids).

The sections of the training were structured as follows:

1. Explanation of related problem/topic from the nursing work life and the goal of the session.
2. Exercises for movement and body perception in the work process (e.g., standing on Airex cushion while a partner tries to get one off-balance, moving partner from lying to a sitting position with several variations of body positions).
3. Reflections of movement experiences and movement optimizations (trainer asks specific questions, e.g., the changing body position, and encourages different movement patterns).
4. Instructions for independent compensation exercises (e.g., side planks, lunges, rowing with resistance band) [20, 30, 31].

**Table 2:** Exercise examples of training sessions for the ergonomics and posture training

Training components	Session	Content and example exercises
General	All	At the beginning of each session, participants will be welcomed. The session and its related problem/topic from the nursing work life will be explained and the goal for the respective session. After the introduction, exercises and leading questions will be dealt with
Work organization	1	Change the workplace to minimize bodily strain Compensation exercise: relaxation of shoulder and neck area (pull up shoulders, hold, release them), 10–15 repetitions
Standing & positioning	2–3	Standing on an Airex cushion with different stances while a partner tries to get one off balance, Lift weight while standing with varying stances on an Airex cushion Compensation exercise: side plank, 2 × until fatigue; standing scale, 3 × for 10 s on each side
Working on the care bed	4–5	Getting off the floor with and without a partner, Lift weight in different angles from the body Compensation exercise: lunges, 3 × 10 per leg; overhead press, 3 × 10 per arm
Transfer situations	6–8	Move partner from a lying to a sitting position with several variations of own or partner’s body position, Lift partner from one to another chair with several variations of body posture, chair positions, etc., Lifting weights with rotation Compensation exercise: rowing with a resistance band, 3 × 10–15 repetitions; deadlift with a resistance band, 3 × 10–15 repetitions; “picking apples”, 3 × for 15 s
Nursing aids and summary	9–10	Lifting weights with aid (e.g., rope) Compensation exercise: wall slides, 3 × 10–15 repetitions

The movement experience consisted of three components: body awareness, recognizing dysfunctional movements, and understanding positive and negative work behavior. All three components were reflected and adapted to the working conditions. The repeated implementation and testing in the work situation should lead to positive attitudes, intentions, and behaviors. Afterward, participants should be able to independently recognize and influence health-related resources and potential dangers.

The strength training took place once a week for 45–60 min, over twelve weeks (Table 3). The sessions were divided into four parts:

1. 5–10 min warm-up and mobilization (e.g., slow aerobic and range of motion exercises for upper ankle joint, hip joint, thoracic spine, shoulder joint, and wrist joint).
2. 10–15 min coordination with theoretical knowledge about conscious movement execution and targeting of muscles and exercises. The goal is to enable participants to reflect and improve their posture and movement patterns (e.g., rotating the tibia around the foot, first in sitting, then in standing).
3. 30–40 min strength exercises (e.g., progressive upper- and lower body exercises, partly with additional resistance band).
4. 5–10 min relaxation (e.g., breathing exercises, self-massage, static stretching).

**Table 3:** Exercise examples of training sessions for strength and resistance training

Training components	Exercise examples		
Mobilization and Warm-up	Exercises for the mobilization of upper ankle joint, hip joint, thoracic spine, shoulder joint, and wrist joint		
Coordination	Imparting theoretical knowledge about conscious movement execution and targeting of muscles Exercises for: Feet (e.g., rotation of the tibia around the foot first in sitting, then in standing position), Hip (e.g., cat-cow), Spine (e.g., round and straighten up the back vertebra by vertebra), Shoulder blades (e.g., breathing into the shoulder blades), Head (e.g., push the head back and forth)		
	Level 1 Week 1–4	Level 2 Week 5–8	Level 3 Week 9–12
Circuit strength training	Deadlift, 2 × 5 repetitions Hip thrust, hold 2 × for 60 s Sit Ups, 2 × for 60 s Lunges, 2 × for 30 s on each side	Deadlift, 2 × 5 repetitions One-legged hip thrust, hold 2 × for 30 s on each side Sitting rotation, 2 × for 60 s Step Up, 2 × for 30 s on each side	Deadlift with a partner, 2 × 5 repetitions Standing scale, hold 2 × for 30 s on each side Lying leg rotation, 2 × for 60 s Split Squad, 2 × for 30 s on each side
Relaxation	Static stretching, self-massage (myofascial release) with, e.g., a tennis ball, breathing exercises, progressive muscle relaxation		

Strength training was divided into three phases (four weeks per phase) with a progression achieved by adjusting the exercises’ difficulty, and intensity/range of motion. The intensity of the exercises was planned to be moderate (5–6 on Borg CR10 Scale [32]).

The program was conducted by certified exercise scientists or physiotherapists trained to work according to a standardized manual. Based on participants’ perceived exhaustion, they could take individual breaks or perform a lighter exercise, simplified by trainers. The interventions took place at the nursing home facilities during working hours in the transition period from early to late shift.

### Statistical methods

All analyses were performed with SPSS Version 27 (IBM SPSS Statistics for Windows, Armonk, NY, USA), with the alpha level set to .05. Descriptive statistics was used to determine the frequency, mean, and standard deviation of each variable. In addition, a one-way Analysis of Variance tested the differences in baseline characteristics.

Linear Mixed Models, fitted with an unstructured covariance matrix, were used to investigate intervention efficacy. The outcomes were assessed for the impact of time. All analyses were performed using fixed effects for group and random intercept per subject. Afterward, a one-way Analysis of Variance was calculated to analyze time point comparisons. Frequency analysis of termination criteria was done by Chi^2^- Tests.

At the end of the trial, we conducted an intention-to-treat analysis with the whole group (including the 26 dropouts) using Linear Mixed Models to avoid the risk of unbalanced groups.

## Main analyses of the outcomes

**Fig. 2:**
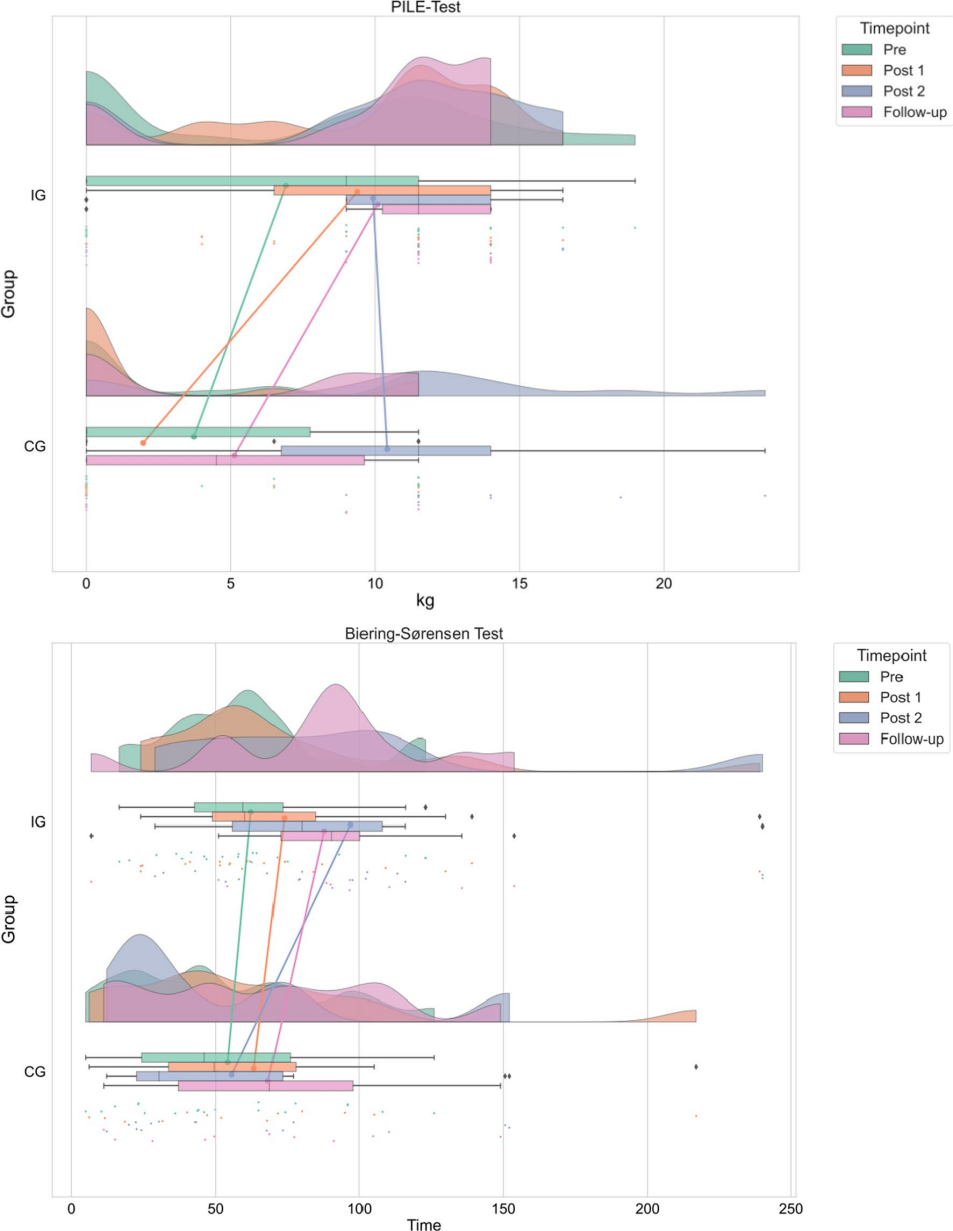
Interactions and main-effects PILE-Test and Biering-Sørensen-Test visualized in cloud plot, rain plot, box plot and line plot

Table 4 reports the descriptive values and statistics of the measurement points (Pre, Post 1, Post 2, Follow-up). The interactions and main effects for the PILE-Test and Biering-Sørensen-Test are illustrated in Fig. 2.

**Table 4:** Descriptive values and linear mixed models statistics

	Pre	Post 1	Post 2	Follow-up	Estimates (SD)	Statistics	*p*
	Mean (SD)					95% CI’s	
PILE-test (kg)							
*Lower upper*							
IG	6.9 (6.5)	9.4 (5.1)	9.9 (5.8)	10.1 (5.2)	4.594 (1.587)	1.378 7.810	.006*
CG	3.7 (5.0)	2.0 (4.2)	10.4 (7.4)	5.1 (5.6)			
*Biering-Sørensen-Test (time)*							
IG	62.3 (30.0)	73.8 (50.3)	96.9 (64.5)	87.7 (38.6)	7.949 (9.866)	− 12.281 28.179	.427
CG	54.2 (35.0)	63.3 (53.4)	55.6 (47.6)	68.3 (43.3)			
*VAS (score)*							
IG	1.2 (1.6)	1.0 (1.7)	1.1 (1.7)	0.9 (1.6)	− 1.011 (0.482)	− 1.987 − 0.034	.043*
CG	2.1 (2.2)	2.7 (2.9)	2.2 (2.2)	1.1 (1.9)			
*ODI (%)*							
IG	7.8 (6.2)	6.6 (9.0)	5.0 (5.2)	6.5 (6.9)	− 4.577 (2.524)	− 9.688 0.533	.078
CG	12.8 (9.9)	10.5 (8.8)	11.5 (11.7)	11.8 (10.4)			

*PILE Test* Progressive Isoinertial Lifting Evaluation, *VAS* Visual Analog Scale, *ODI* Oswestry Disability Index, *IG* intervention group, *CG* control group

*p < 0.05

The analysis showed significant differences between the IG and the CG in the lifted weight during the PILE-Test. Moreover, the nurse's perception of LBP intensity on the VAS was reduced in the IG (Table 4). Group comparisons revealed significant differences at the second measurement time point (Post 1) of the PILE-Test (F_(1,34)_ = 21.070, *p* < 0.001) and the VAS (F_(1,34)_ = 5.021, *p* = 0.032). At the same time, the termination criteria for the PILE-Test changed (Table 5). The IG recorded fewer terminations due to thoracic spine hyperkyphosis (Chi^2^ = 9.531, *p* = 0.002) and more terminations due to muscular fatigue (Chi^2^ = 10.413, *p* = 0.001) than the CG after participation in ergonomics training. There were no differences concerning the test time regarding the Biering-Sørensen-Test and the functional impairment caused by back pain (ODI).

**Table 5:** Termination criteria for the PILE-Test

Termination criteria	%							
	Pre		Post 1		Post 2		Follow-up	
	IG	CG	IG	CG	IG	CG	IG	CG
85% of HR max	18.2	25.0	23.8	13.3	28.6	16.7	9.1	25.0
50% body weight	0	0	0	0	0	0	0	0
20-s interval	0	5.0	9.5	0	0	25.0	36.4	12.5
Muscular fatigue	18.2	5.0	42.9*	0	35.7	25.0	36.4	12.5
Thoracic spine hyperkyphosis	54.5	65.0	19.1*	86.7	28.6	25.0	18.2	50.0
Pain	9.1	0	4.8	0	7.1	8.3	0	0

*IG* intervention group, *CG* control group

*p < 0.05

The intention-to-treat analysis showed no significant differences between the IG and the CG concerning the LBP intensity on the VAS (95% CI − 0.49 to 1.66, *p* = 0.281).

Overall, the participants were optimistic about the program. The participants enjoyed the content, liked the exercises, had fun, and wanted to learn more about personal health promotion (Table 6).

**Table 6:** Evaluation of the ergonomics and posture training (n = 32) and strength training (n = 31)

Questions	Yes, I agree (%)		No, I disagree (%)		No response (%)	
	*ep*	*st*	*ep*	*st*	*ep*	*st*
I enjoyed the training content	95.5	93.6	0	6.5	4.5	0
I liked the exercises	93.2	93.6	4.5	6.5	2.3	0
I think the exercises are useful	95.5	96.8	2.3	0	2.3	3.2
I learned something new	88.7	83.9	11.4	12.9	0	3.2
I remembered previous teaching	70.5	67.7	25.0	25.8	4.5	6.5
I had fun during the exercises	90.9	96.8	6.8	0	2.3	3.2
The exercises were related to my everyday life	86.3	77.4	6.8	19.4	6.8	3.2
I was able to execute the exercises	86.4	96.7	11.4	0	2.3	3.2
The trainers expressed themselves in an understandable way	86.4	93.5	11.4	3.2	2.3	3.2
The training was too short	75.0	71.0	20.5	29.0	4.5	0
The amount of time of the training was appropriate	79.5	90.3	13.6	6.5	6.8	3.2
I would like to learn more about personal health promotion	90.9	80.6	9.1	12.9	0	6.5

## Discussion

This RCT aimed to investigate the effectiveness of a tailored multicomponent intervention (combination of ergonomics and strength training) to improve lifting behavior, strength endurance of the lumbar extensors, LBP, and functional impairment caused by back pain for elderly care nurses. Additionally, participants’ adherence to the intervention was assessed.

The results showed positive effects on IG nurses’ lifting performance, illustrated by higher lifted weight with concomitant changes in termination criteria. Furthermore, the analysis revealed significant differences between both groups in back pain (after IGs’ participation in ergonomics training). The differences were found at the second measurement time point. However, the effect on LBP did not remain within the intention-to-treat analysis.

The further evaluation revealed no significant differences at any measurement time point concerning the strength endurance of the lumbar extensors and the functional impairment caused by LBP. As expected, participants’ adherence showed positive ratings.

Regarding the lifting performance and behavior as one common risk factor for increasing LBP, the analysis of the PILE-Test revealed significant improvements in the IG after participation in ergonomics training. This effect in the IG remained similarly positive after participating in strength training. Additionally, the CG showed comparable improvements after their ergonomics intervention, including the same training contents. In line with Ewert and colleagues [16], our data support that ergonomics training successfully increases lifting performance for both groups.

Notably, the groups also differed in their quality of movement execution. The IG demonstrated a simultaneous increase in terminations due to muscular fatigue in addition to reduced terminations due to thoracic spine hyperkyphosis. We assume a reason might be the increased lifted weight during the test and individuals' low physical capacity [6]. However, the differences were only present at the second measurement point, indicating that both groups benefited equally from the training. In line with Wollesen and colleagues [20], ergonomic training reduced dysfunctional lifting behavior successfully. Therefore, we suggest that the program is suitable to reduce a major risk factor of LBP. Moreover, we can summarize that the training content was successfully adapted to the nursing field and promises to be useful for further implementation in other care settings.

In addition, the IG increased their Biering-Sørensen-Test time by approximately 23 s after participating in the strength training, while test time of the CG decreased. However, these observations failed to be significant. We assume that the results are caused by interindividual differences between the nurses’ strength endurance, which are reflected in the standard deviations (cf. Table 2). Our results align with Stevens and colleagues [14], even though they measured subjectively perceived strength. The lack of significant group differences can probably be attributed to methodological problems we observed, such as motivation, tolerance of the discomfort of fatiguing muscles or pain, or fear of pain [29]. This observation suggests that another test is needed to obtain valid data (e.g., electromyography).

Moreover, the duration of the strength training might have been insufficient to increase strength. The nurses in our study were analyzed when they reached at least a 70% participation rate, which corresponds to participation in at least nine units over nine weeks. A recent meta-analysis recommends training over 12 to 16 weeks to gain significant improvements in strength. Furthermore, according to the specific training stimulus [33], criteria of load control, stimulus scope, and stimulus density must be considered to generate adaptation effects. In our study, despite dividing the strength training into three phases with progression, the degree of difficulty had to be increasingly adapted individually to the participants. Therefore, the overly high level of difficulty resulted in simplifying the exercises, leading to inadequate progression control. Overall, future strength interventions should be performed for extended periods with a higher stimulus density and include exercises with a lower intensity and slower progression. This highlights the need to address nurses’ time constraints and offer alternate dates for training sessions that could not attend.

The analysis of the LBP within the VAS revealed significant differences between both groups. Participation in the ergonomics training resulted in the same Level of LBP in the IG, whereas pain slightly increased in the CG. As a clinically significant change does not occur until a value of 20 mm, the results should be interpreted with caution [34]. Nevertheless, the further aggravation of the staff shortage may have affected results in the CG [9]. Unfortunately, this effect did not remain in the intention-to-treat analysis. One might assume that the reason, again, was the heterogeneity of the recorded data, exposed by high standard deviation. However, the participants failed to follow-up were not significantly different from the considered participants concerning baseline characteristics and primary and secondary outcomes studied.

Future studies should verify the pain situation over at least 17–20-weeks, consistent with the literature [33]. Nevertheless, we suppose that our tailoring, the individual adaptation of the training content to the conditions of the nursing home facility, and the movement experiences, including body awareness, recognition of dysfunctional movements, and understanding positive and negative work behavior, might affect back pain positively. This highlights the need for interventions, considering the bottom-up approach, taking work-related burdens, wishes, barriers, and the facility’s condition into account [1].

Surprisingly, the intensity of LBP in the IG and CG was relatively low, compared to the high prevalence of LBP (around 50%) in this target group which is, additionally, repeatedly demonstrated in the literature [6, 35]. The discrepancy might indicate a lack of ability of the nurses to discriminate between the presence of pain and assessing the pain intensity which is associated with a possible underestimation of the severity of LBP. As a result, nurses reported low functional impairment caused by back pain. The evaluation of the ODI and the classification of the percentages showed a minimal disability in both groups and no significant differences in the study [29]. However, the intervention period may not have been long enough to reduce functional impairment and should be considered more closely in future investigations. Overall, the low intensity in LBP and the low functional impairment of all included nurses may also indicate that only those who already live health-consciously and suffer less from pain and functional impairment were motivated to participate in the intervention. For this reason, we recommend conveying education about the importance, the mode of action, and the effects of health-promoting interventions in future research to motivate the non-participating nurses.

Regarding the participation, it is newsworthy to report that the intervention program achieved a positive adherence. The participants of the CG determined the high dropout rate. Within the participants that joined the program regularly, the dropout quote was only 24%. According to our results, the nurses equally accepted and tolerated the intervention. Nurses regularly participated in the intervention despite the barriers, such as time pressure. This highlights the need for the participatory approach in the process and the tailored structure of the training with the individually adapted intensity of the exercises [20].

## Limitations

Next to the study’s strengths, some limitations need to be addressed. The participants took part in the study during working hours. Therefore, time pressure, lack of time, or motivation could have influenced the data assessment. Moreover, the LBP and functional impairment were assessed with self-administered questionnaires and scale. This may have led to a possible underestimation of the severity of LBP and disability due to a lack of ability for discrimination between the presence of pain and the assessment of the pain intensity.

The results were not controlled for the duration of employment, the type of employment (e.g., full time, part-time), or the activities during leisure time which might be a relevant factor for burdens and strains. Moreover, the high level of difficulty in back fitness and the resulting simplification of the exercises could have led to the progression not being sufficiently controlled. Finally, the dropout rate of 37% was high. However, considering the difficulties in implementing a randomized controlled study in elderly care, the number of participants achieved can be considered a success and fulfilled the prior calculated power.

## Conclusion

The intervention reported in this study showed positive effects on lifting performance, ergonomic behavior, and LBP. Moreover, the multidimensional approach of the BASE concept led to a positive adherence as it includes the specific integration of the expected work of the target group. Thus, we conclude that a multicomponent intervention tailored to the target group, taking aspects such as movement experiences and strength training into account, can positively affect nurses. The results of this study are essential to derive more specific recommendations for effective prevention in nursing home facilities. As the number of longitudinal studies with multicomponent interventions tailored for elderly care nurses is rare, the positive results can be completed in future studies with additional strategies for pain reduction, ratio prevention, and work situations to reduce burden of the nurses further.

## Data Availability

The datasets generated and analyzed during the study are available from the corresponding author on reasonable request.
